# Pharmacological inhibition of MALT1 (mucosa-associated lymphoid tissue lymphoma translocation protein 1) induces ferroptosis in vascular smooth muscle cells

**DOI:** 10.1038/s41420-023-01748-9

**Published:** 2023-12-15

**Authors:** Binjie Yan, Darrell Belke, Yu Gui, Yong-Xiang Chen, Zhi-Sheng Jiang, Xi-Long Zheng

**Affiliations:** 1 Departments of Biochemistry & Molecular Biology and Physiology & Pharmacology, Cumming School of Medicine, University of Calgary, Calgary, AB T2N 4Z6 Canada; 2 Institute of Cardiovascular Disease, Key Laboratory for Arteriosclerology of Hunan Province, Hengyang Medical School, University of South China, Hengyang, Hunan China; 3 Department of Cardiac Sciences, Cumming School of Medicine, University of Calgary, Calgary, AB Canada

**Keywords:** Cell death, Biochemistry

## Abstract

MALT1 (mucosa-associated lymphoid tissue lymphoma translocation protein 1) is a human paracaspase protein with proteolytic activity via its caspase-like domain. The pharmacological inhibition of MALT1 by MI-2, a specific chemical inhibitor, diminishes the response of endothelial cells to inflammatory stimuli. However, it is largely unknown how MALT1 regulates the functions of vascular smooth muscle cells (SMCs). This study aims to investigate the impact of MALT1 inhibition by MI-2 on the functions of vascular SMCs, both in vitro and in vivo. MI-2 treatment led to concentration- and time-dependent cell death of cultured aortic SMCs, which was rescued by the iron chelator deferoxamine (DFO) or ferrostatin-1 (Fer-1), a specific inhibitor of ferroptosis, but not by inhibitors of apoptosis (Z-VAD-fmk), pyroptosis (Z-YVAD-fmk), or necrosis (Necrostatin-1, Nec-1). MI-2 treatment downregulated the expression of glutathione peroxidase 4 (GPX4) and ferritin heavy polypeptide 1 (FTH1), which was prevented by pre-treatment with DFO or Fer-1. MI-2 treatment also activated autophagy, which was inhibited by Atg7 deficiency or bafilomycin A1 preventing MI-2-induced ferroptosis. MI-2 treatment reduced the cleavage of cylindromatosis (CYLD), a specific substrate of MALT1. Notably, MI-2 treatment led to a rapid loss of contractility in mouse aortas, which was prevented by co-incubation with Fer-1. Moreover, local application of MI-2 significantly reduced carotid neointima lesions and atherosclerosis in C57BL/6J mice and apolipoprotein-E knockout (ApoE^−/−^) mice, respectively, which were both ameliorated by co-treatment with Fer-1. In conclusion, the present study demonstrated that MALT1 inhibition induces ferroptosis of vascular SMCs, likely contributing to its amelioration of proliferative vascular diseases.

## Introduction

The mucosa-associated lymphoid tissue lymphoma translocation protein 1 (MALT1) was initially identified in lymphocytes and is a human paracaspase protein that serves as an adapter downstream of protein kinase C [1]. MALT1 paracaspase forms a signalosome complex with caspase recruitment domain family member 11 (CARD11 or CARMA1) and B cell CLL/lymphoma 10 (BCL10). This complex mediates the activation of the IκB kinase (IKK) complex and the transcription of nuclear factor kappa-light-chain-enhancer of activated B (NF-κB)-induced genes, leading to activation and proliferation in immune cells [2]. In non-immune cells, CARD recruited membrane-associated protein 3 (CARMA3) can substitute for CARMA1 to assemble a CARMA3-BCL10-MALT1 (CBM) signalosome [3].

Deficiencies in MALT1 have been associated with various inflammatory conditions and related pathologies due to impaired T cell and antibody responses. These conditions include dermatitis [4] and osteoporosis [5]. MI-2, a specific MALT1 chemical inhibitor [6], is currently under investigation for its potential in treating various diseases [7–11].

Several MALT1 substrates, such as monocyte chemotactic protein-induced protein 1 (MCPIP1), cylindromatosis (CYLD), BCL10, and the ubiquitin-editing enzyme A20, have been identified to date [12–14]. However, our understanding of the expression and role of MALT1 and its targeted substrates in the cardiovascular system remains limited. To date, there has been minimal research regarding their potential involvement in cardiovascular diseases, including atherosclerosis [3, 15, 16] and heart ischemia/reperfusion (I/R) [17].

In endothelial cells, thrombin triggers MALT1 to proteolytically cleave CYLD, leading to microtubule disruption and a series of events promoting tissue inflammation [16]. Furthermore, it has been reported that MALT1 paracaspase degrades the MCPIP1 protein in endothelial cells. The inhibition of MALT1 protease activity by MI-2 suppresses the expression of vascular cell adhesion molecule 1 (VCAM-1) on endothelial cells both in vitro and in vivo. MI-2 also inhibits monocyte adherence to activated endothelial cells, an effect dependent on increased expression of MCPIP1 but not on NF-κB signaling [15].

In vascular SMCs, the angiotensin II type 1 receptor (AT1R) employs the CARMA3-containing CBM signalosome for NF-κB activation and induction of pro-inflammatory signals, suggesting a potential role for MALT1 in angiotensin II-associated cardiovascular diseases [3]. However, the roles of MALT1 in the cardiovascular system remain to be elucidated.

To date, reports suggest that pharmacological inhibition of MALT1 leads to apoptosis in various cell types [18–21]. Jiang et al. recently demonstrated that MI-2 inhibition of MALT1 reduces I/R-induced myocardial ferroptosis through the enhancement of the nuclear factor erythroid-2/solute carrier family 7 member 11 (Nrf2/SLC7A11) pathway [17], suggesting a role for MALT1 in the occurrence of ferroptosis.

Ferroptosis is a type of programmed cell death dependent on iron [22], previously reported in vascular SMCs [23–25]. The induction of ferroptosis was reported to result in SMC phenotypic conversion and the acceleration of neointima formation in mouse carotid arteries [23]. In addition, ferroptosis induction in SMCs by cigarette smoke extract has been shown to cause loss of contractility of aortic tissues [26].

In the current study, we found that MI-2 treatment induces ferroptosis of cultured vascular SMCs through the activation of autophagy, and results in a rapid loss of contractility in mouse aortic rings ex vivo. We also found that local application of MI-2 inhibits neointima formation and carotid arterial atherosclerosis. Our results suggest that targeting MALT1 could potentially become a therapeutic approach for the treatment of proliferative vascular diseases.

## Results

### Treatment with MI-2 induces ferroptotic cell death in aortic SMCs

**Fig. 1:**
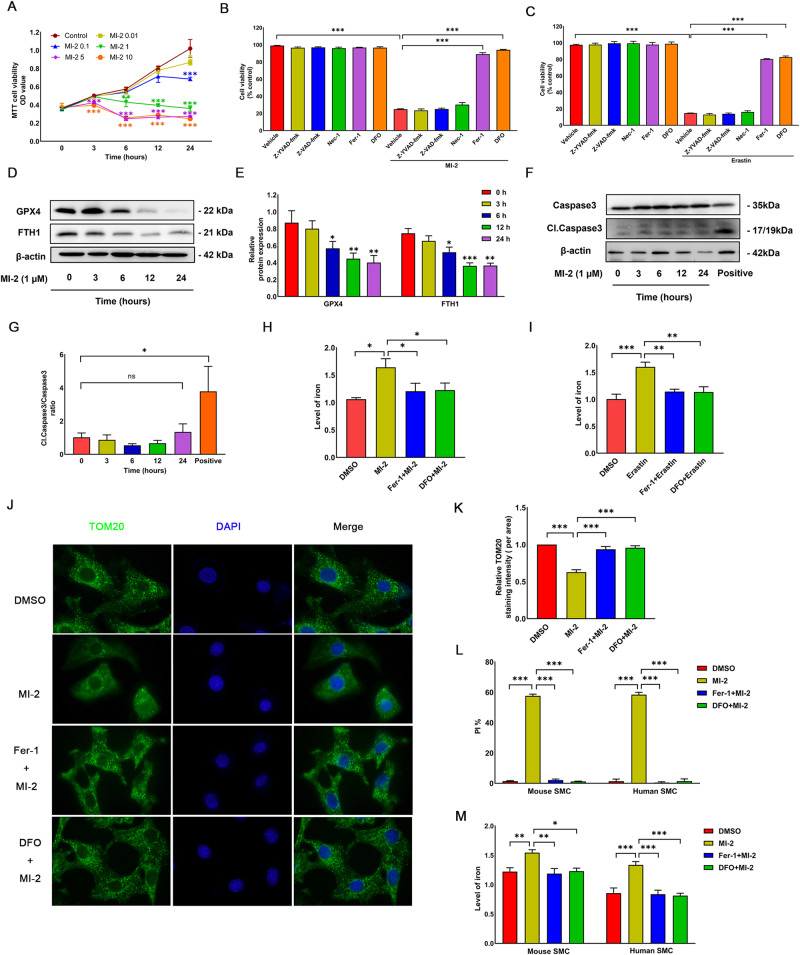
MI-2 induces ferroptosis in rat aortic smooth muscle cells (SMCs). **A** Cell viability. SMCs were treated with MI-2 for the indicated concentrations and times, followed by the MTT assay. *p < 0.05; **p < 0.01; ***p < 0.001; *n* = 4. **B**, **C** SMC death induced by MI-2 (**B**) or erastin (**C**) was rescued by Fer-1 and DFO. SMCs were treated with MI-2 (1 µM) or erastin (5 µM) for 24 h with and without various inhibitors for cell death, followed by the MTT assay. ***p < 0.001; *n* = 4. **D**, **E** MI-2 time-dependently reduced the protein expression of GPX4 and FTH1. SMCs were treated with MI-2 (1 µM) for the indicated times, followed by protein extraction and WB assays (**D**, representative WB images; **E**, cumulative data showing expression levels of GPX4 and FTH1 relative to β-actin). *p < 0.05; **p < 0.01; ***p < 0.001; *n* = 3. **F**, **G:** MI-2 did not cause cleavage of caspase 3. Cultured SMCs were treated with MI-2 (1 µM) for the indicated times up to 24 h or with 2-methoxyestradiol (2-ME, 1 µM) for 24 h as a positive control (Positive), followed by WB assays for both total and cleaved caspase 3 (**F**, representative WB; **G**, cumulative data). *p < 0.05; *n* = 3. **H**, **I** MI-2 (**H**) and erastin (**I**) increased cellular iron levels, which Fer-1 and DFO inhibited. SMCs were treated with MI-2 (1 μM, 6 h) or erastin (5 μM, 24 h) in the presence or absence of Fer-1 or DFO, followed by measuring iron levels using the Iron Colorimetric Assay Kit. *p < 0.05; **p < 0.01; ***p < 0.001; *n* = 3. **J**, **K** MI-2 treatment disrupted mitochondrial structure, which Fer-1 and DFO rescued. SMCs were treated with MI-2 (1 µM) with or without the presence of Fer-1 or DFO for 6 h, followed by immunostaining for TOM20 with the nuclei counterstained with DAPI (**J** representative immunofluorescence micrographs; **K** cumulative data showing the fluorescence intensity of TOM20). ***p < 0.001; *n* = 3. **L**, **M** Cumulative data showing primary cultured mouse and human aortic SMCs treated with MI-2 (1 µM) in the absence or the presence of Fer-1 or DFO for 6 h, followed by PI-Hoechst co-staining (**L**) or the Iron Colorimetric Assay for intracellular iron levels (**M**). *p < 0.05; **p < 0.01; ***p < 0.001; *n* = 3.

To explore the role of MALT1 in vascular functions, we examined the effects of various concentrations of the MALT1 inhibitor MI-2 (0, 0.01, 0.1, 1, 5, and 10 μM) on the viability of cultured SMCs via the MTT assay. The application of MI-2 at 0.1 μM led to a significant reduction in SMC viability after 24 h (Fig. 1A). At concentrations of 2.5 or 10 μM, MI-2 reduced cell viability even as early as 3 h. Moreover, at 1 μM, MI-2 caused a time-dependent decrease in cell viability. Importantly, the cell death effects induced by MI-2 were significantly mitigated by co-treatment with DFO (100 µM) or Fer-1 (5 µM), both of which are ferroptosis inhibitors, but not by Z-YVAD-fmk (10 µM), Z-VAD-fmk (25 µM), or Nec-1 (10 µM), which are inhibitors for apoptosis, pyroptosis, and necrosis, respectively (Fig. 1B). Morphologically, MI-2 treatment induced the rounding of cultured SMCs, which was inhibited by blocking ferroptosis (Supplementary Fig. S1A). Supportively, erastin, a known ferroptosis inducer, triggered similar cell death (Fig. 1C) and morphological changes (Supplementary Fig. S1B). These effects were also reversed by DFO and Fer-1 but not by the other inhibitors for apoptosis, pyroptosis, and necrosis (Fig. 1C and Supplementary Fig. S1B).

Given that ferroptosis represents an iron-dependent, non-apoptotic form of cell death distinct from apoptosis [27], and that MALT1 inhibition has been reported to induce apoptosis, we further characterized MI-2-induced ferroptosis by evaluating the expression of ferroptosis- and apoptosis-related proteins. Our results demonstrated that treatment with MI-2 at 1 μM decreased the protein expression levels of GPX4 and FTH1 in a time-dependent manner (Fig. 1D, E); however, it did not increase cleaved caspase 3 levels (Fig. 1F, G). This further confirmed the presence of ferroptotic cell death rather than apoptosis. Notably, we observed that MI-2-induced ferroptosis occurred after a 6-h treatment. Thus, we used the 6-h time point for most of our subsequent experiments. Consistent with our findings, treatment with MI-2 (Fig. 1H) or erastin (Fig. 1I) significantly increased intracellular iron levels, which were reversed by co-treatment with Fer-1 or DFO. Given that the induction of ferroptosis is known to cause mitochondrial dysfunction, characterized by small mitochondria with condensed mitochondrial membrane densities [28], we stained cultured SMCs for mitochondrial import receptor subunit TOM20 after MI-2 treatment to visualize mitochondrial morphologies. As shown in Fig. 1J, K, treatment with MI-2 induced a diffused pattern of TOM20 staining with decreased fluorescence density, which was reversed by Fer-1 and DFO. Moreover, treatment with MI-2 also induced ferroptotic cell death in both human and mouse aortic SMCs (Fig. 1L, M and Supplementary Fig. S2A, B). Our data suggest that MI-2 induces ferroptosis in cultured vascular SMCs.

### Autophagy activation is involved in MI-2-induced ferroptosis

Next, we investigated the potential mechanism by which MI-2 triggers ferroptosis. Extensive evidence suggests that excessive activation of autophagy induces ferroptotic cell death caused by various ferroptosis activators across different cell types [29–32]. To explore the involvement of autophagy in MI-2-induced ferroptosis of cultured aortic SMCs, we co-treated SMCs with inhibitors of late-stage autophagy, bafilomycin A1 (Baf A1) and chloroquine (CQ), and then carried out propidium iodide (PI) staining for cell death.

**Fig. 2:**
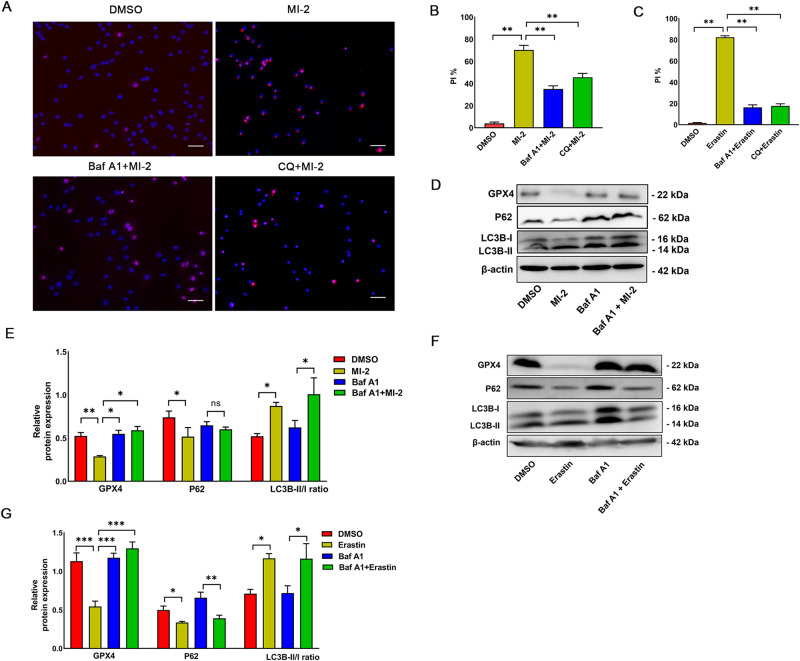
MI-2 induces ferroptosis in vascular SMCs through autophagy activation. **A**–**C**: 1 µM MI-2- or 5 µM erastin-induced cell death was inhibited by bafilomycin A1 (Baf A1, 100 nM) or chloroquine (CQ, 50 µM). Rat aortic SMCs were treated with MI-2 in the presence or absence of Baf A1 (100 nM) or CQ (50 µM) for 6 h, followed by PI-Hoechst co-staining (**A**, representative micrographs; **B**, cumulative data). Scale bar = 100 μm. **p < 0.01; *n* = 3. **C**: Cumulative data for erastin treatment for 24 h in the presence of Baf A1 (100 nM) or CQ (50 µM). **p < 0.01; *n* = 3. **D**–**G** Baf A1 inhibited autophagy and GPX4 reduction induced by MI-2 (**D**, **E**) or erastin (**F**, **G**). Cultured aortic SMCs were treated with MI-2 (1 µM, 6 h) or erastin (5 µM, 24 h) in the presence or absence of Baf A1 (100 nM), followed by extraction of total cell lysate proteins and WB assays. **D**, **F** Representative WB images. **E**, **G** Cumulative data showing expression levels of GPX4, p62 and LC3B-II/I relative to β-actin. *p < 0.05; **p < 0.01; ***p < 0.001; *n* = 3–4/group.

As shown in Fig. 2A, B, autophagy inhibition significantly reduced cell death induced by MI-2. Biochemically, MI-2 treatment diminished the levels of GPX4 and p62, an autophagic receptor protein, which is degraded in response to autophagy induction (Fig. 2D, E). These responses were partially reversed by Baf A1 (100 nM). We noted a significant increase in autophagic flux, as evidenced by the enhanced conversion of LC3B-I to LC3B-II following MI-2 treatment (Fig. 2D, E). Notably, both Baf A1 and CQ obstructed ferroptosis at earlier time points (6 h), while the inhibitory effect gradually diminished at later time points (after 12 or 24 h), particularly when a higher dose of MI-2 was used. Importantly, we confirmed the same trend in biochemical protein changes induced by erastin (5 µM) in cultured aortic SMCs, as depicted in Fig. 2C, F, G and Supplementary Fig. S3. Our findings suggest a critical role for autophagy activation in MI-2-induced ferroptosis in aortic SMCs.

### Mouse aortic SMCs deficient in Atg7 are resistant to MI-2-induced ferroptosis

**Fig. 3:**
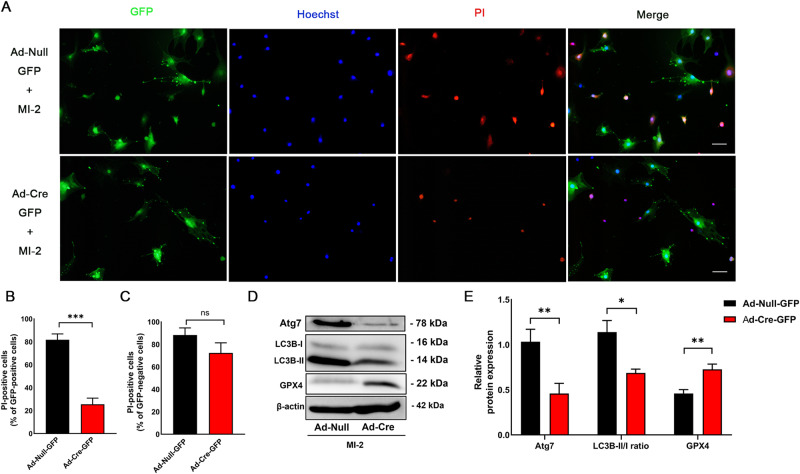
Mouse aortic SMCs deficient in Atg7 are resistant to MI-2-induced ferroptosis. Mouse aortic SMCs were cultured from Atg7^fl/fl^ mice as described in the Methods. SMCs at passage 3 were transduced with Cre recombinase adenovirus (Ad-Cre) or adenovirus (Ad-Null) for 48 h, followed by treatment with MI-2 (1 µM) for 6 h and subsequent PI-Hoechst co-staining and WB assays. **A** Representative fluorescent micrographs showing PI-Hoechst co-staining in primarily cultured mouse aortic SMCs treated with MI-2 (1 µM, 6 h) after incubation with Ad-Cre-GFP or Ad-Null-GFP. Scale bar = 100 μm. **B**, **C** Cumulative data showing the proportion of PI-positive cells in GFP-positive cells with or without Cre expression (**B**); and the proportion of PI-positive cells in GFP-negative cells (**C**). ***p < 0.001; ns not significant; *n* = 5–7/group. **D**, **E** Representative WB images (**D**) and cumulative data (**E**) showing Atg7, LC3B-II/I, and GPX4 levels relative to β-actin. *p < 0.05; **p < 0.01; *n* = 4–6/group.

To further substantiate the role of autophagy in MI-2-induced ferroptosis, we used primary cultured mouse aortic SMCs deficient in Atg7 to evaluate the ferroptotic effects of MI-2. As outlined in the Methods, primary aortic SMCs were cultivated from Atg7^fl/fl^ mice. Cells from the third passage were transduced with an adenovirus expressing Cre recombinase (Ad-Cre-GFP) or Ad-Null-GFP alone, as we previously described [33]. Atg7 deficiency in primary mouse aortic SMCs was confirmed by Western blot (WB) analysis (Fig. 3D, E). Mouse aortic SMCs with Atg7 deficiency (Ad-Cre-GFP) exhibited notably less ferroptotic cell death in response to treatment with MI-2 (Fig. 3A, B), compared with control SMCs expressing GFP only (Ad-Null-GFP), as indicated by PI-Hoechst staining. However, the GFP-negative cells, which were not infected by either virus, had the same PI positive rates in both groups (Fig. 3A, C). For biochemical confirmation, the expression of GPX4 protein was substantially reduced after MI-2 treatment in the Atg7-deficient group, accompanied by an increase in the LC3B-II/I ratio (Fig. 3D, E). In summary, the inhibition of autophagy by knocking out Atg7 mitigates MI-2-induced ferroptosis, supporting the role of autophagy activation in MI-2-induced ferroptosis in vascular SMCs.

### MI-2-induced autophagy is associated with the Akt/mTOR/p70 S6K pathway

**Fig. 4:**
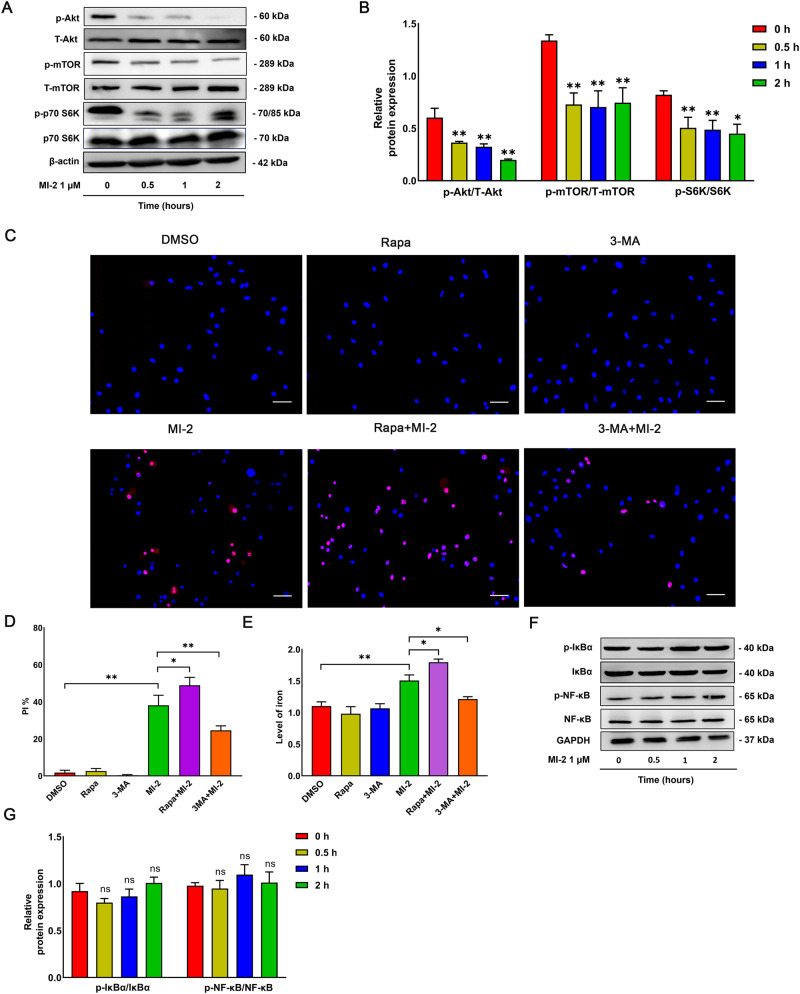
MI-2-induced autophagy is associated with the Akt/mTOR/p70 S6K pathway but not the NF-κB pathway. Cultured aortic SMCs were treated with MI-2 (1 µM) for the indicated times, followed by extraction of total cell lysate proteins and WB assays. **A**, **B** Representative WB images (**A**) and cumulative data (**B**) showing the relative levels of p-Akt, T-Akt, p-mTOR, mTOR, p-p70 S6K, p70 S6K, and β-actin. *p < 0.05; **p < 0.01; *n* = 3–5/group. **C**–**E** Representative images (**C**) and cumulative data (**D**) showing rat primary SMCs treated with MI-2 for 6 h in the presence of rapamycin (Rapa; 100 nM) or 3-MA (50 µM), followed by PI-Hoechst co-staining and Iron Colorimetric Assay for intracellular iron levels (**E**). Scale bar = 100 μm. *p < 0.05; **p < 0.01; *n* = 3. **F**, **G** Representative WB images (**F**) and cumulative data (**G**) showing p-IκBα, IκBα, p-NF-κB, NF-κB, and β-actin. ns not significant; *n* = 3.

Given that ferroptosis is also regulated by the protein kinase B (Akt)/mechanistic target of rapamycin (mTOR)/70-kD ribosomal protein S6 kinase (p70 S6K) signaling pathway [34], we assessed whether MI-2-induced autophagy occurs through inhibiting the Akt/mTOR/p70 S6K axis. We observed that MI-2 treatment inhibited Akt phosphorylation and activation, accompanied by the inactivation of mTOR and p70 S6K phosphorylation at site Ser2448 and site Thr389, respectively, at the indicated times (Fig. 4A, B). To further assess the role of the Akt/mTOR/p70 S6K axis in MI-2-induced ferroptosis, we also pre-treated cultured aortic SMCs with 3-methyladenine (3-MA, 50 µM), a phosphoinositide 3-kinase (PI3K) and autophagy blocker, and rapamycin (Rapa, 100 nM), an mTOR inhibitor and autophagy activator. This was followed by PI-Hoechst co-staining and iron level detection. As anticipated, 3-MA notably decreased cell death, while rapamycin, which augments autophagy, promoted cell death (Fig. 4C–E). Given that previous studies have indicated that MALT1 serves as a scaffold protein playing a significant role in the activation of the NF-κB pathway in several cell lines [9, 20, 35, 36], we aimed to determine the activation of the NF-κB pathway. However, in cultured aortic SMCs, MI-2 treatment did not interfere with NF-κB signaling activation as suggested by the lack of changes in both p-IκBα (Ser32/36 site) and p-NF-κB (Ser536) (Fig. 4F, G). Concurrently, pre-treatment with QNZ (EVP4593, 1 µM), an NF-κB inhibitor, did not reverse MI-2-induced ferroptosis (Supplementary Fig. S4). In summary, our results suggest that MI-2-induced autophagy likely results from the inhibition of the Akt/mTOR/p70 S6K axis.

### MI-2 reduces the cleavage of CYLD, one of the substrates of MALT1 activity

**Fig. 5:**
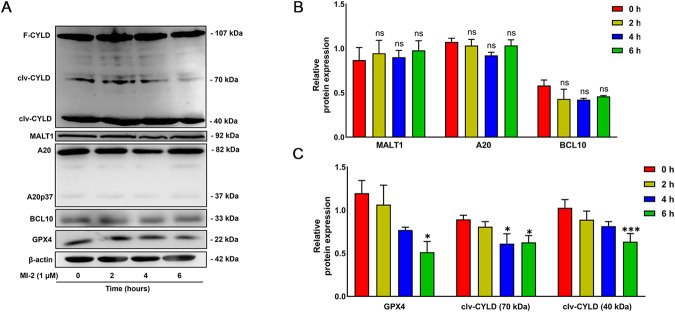
MI-2 treatment reduces the cleavage of CYLD in vascular SMCs. Cultured aortic SMCs were treated with MI-2 (1 µM) for the indicated times, followed by extraction of total cell lysate proteins and WB assays. **A**: Representative WB images showing full-length CYLD (F-CYLD), cleavage CYLD (clv-CYLD, 70 kDa and 40 kDa), MALT1, A20, A20p37, BCL10, GPX4, and β-actin. **B, C** Cumulative data showing F-CYLD, clv-CYLD (70 kDa and 40 kDa), MALT1, A20, BCL10, and GPX4 levels relative to β-actin. *p < 0.05; ***p < 0.001; ns not significant; *n* = 3–5/group.

MI-2 was developed as an irreversible MALT1 protease activity inhibitor [6]. It is well-established that MALT1 cleaves several known substrates, including CYLD, A20, BCL10, and MCPIP1, via its protease activity [12, 13, 15]. To indirectly demonstrate MI-2 inhibition of MALT1 activity, we investigate whether MI-2 treatment reduces the cleavage of MALT1 substrates in cultured vascular SMCs. As shown in Fig. 5A, C, treatment of cultured aortic SMCs with MI-2 (1 μM) significantly reduced the levels of cleaved forms of CYLD (70 and 40 kDa, representing C-terminal and N-terminal fragments, respectively), as previously reported [12], and the levels of GPX4. However, MI-2 treatment did not affect the total expression levels of A20 and BCL10 (Fig. 5A, B). Supportively, there was a low level of the cleaved form of A20 (A20p37), which did not change significantly in response MI-2 treatment. MCPIP1 was not detectable, regardless of the presence or absence of MI-2 (data not shown). It should be noted that there was no significant change in MALT1 protein expression in response to MI-2 treatment, which is consistent with observations from others [15, 37].

### MI-2 induces the loss of vascular contractility, which is reversed by Fer-1

**Fig. 6:**
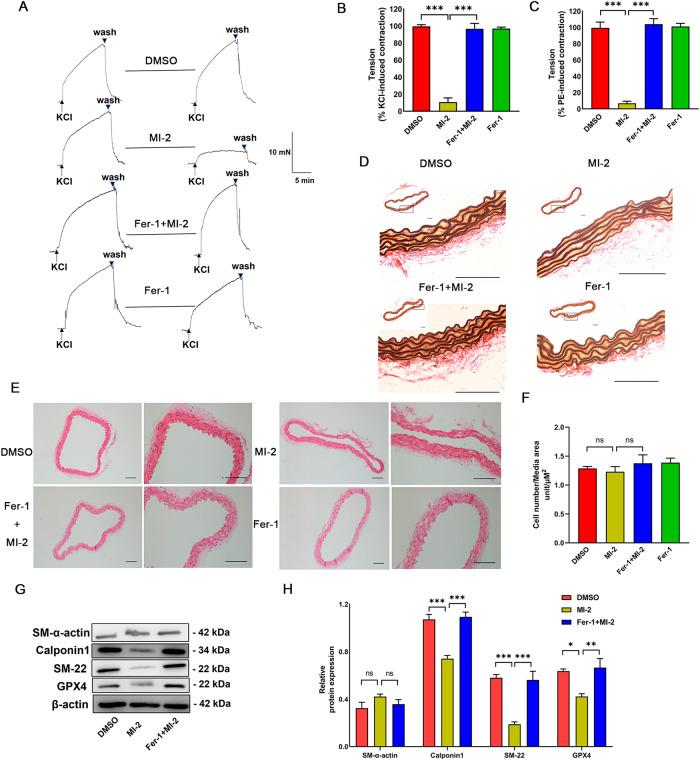
MI-2 results in the loss of vascular contractility ex vivo, which is reversed by Fer-1. Mouse aortic tissues with endothelium removed were isolated for wired myography. Aortic preparations were pre-treated with and without DMSO or Fer-1 (10 µM) for 90 min, followed by MI-2 (2.5 µM) incubation for 90 min, and then adding 50 mM KCl and 1 µM PE. **A** Representative tracings showing KCl responses of tissues before and after treatment with MI-2 (2.5 µM) in the presence of Fer-1 (10 µM) pre-treatment. **B**, **C** Cumulative data for 50 mM KCl- and 1 μM PE-induced contractile responses. ***p < 0.001; *n* = 3. **D**, **E** Mouse aortic tissues after wired myography were fixed and sectioned (5 µm thick) for Elastic Van Gieson (EVG) staining (**D**) and HE staining (**E**) for the nuclei count. Scale bar = 100 µm. **F** Cumulative data showing the ratio of cell number to media area. ns: not significant. **G** Representative WB images showing the expression level of SM-α-actin, Calponin1, SM-22, GPX4, and β-actin. **H** Cumulative data showing SM-α-actin, Calponin1, SM-22, and GPX4 expression levels relative to β-actin. *p < 0.05; **p < 0.01; ***p < 0.001; ns not significant; *n* = 3–4/group.

Previous research has reported that treating aortic tissues with cigarette smoke extract induces SMC ferroptosis, rapidly losing medial vascular SMCs in ex vivo mouse aortas [26]. Therefore, we decided to evaluate the ex vivo effects of MI-2 on the contractility of mouse aortas using wired myography. As depicted in Fig. 6A–C and Supplementary Fig. S5, our data demonstrated that pre-treatment of endothelium-denuded aortic tissues with MI-2 (5 µM) for 90 min significantly inhibited the contractile responses of mouse aortas induced by KCl (50 mM) and PE (1 µM). Crucially, the inhibitory effect of MI-2 on contractility was reversed by co-treatment with the ferroptosis inhibitor, Fer-1. Subsequently, we conducted histological studies on the aortic tissues from the wired myography experiments. In control arterial tissue, elastin fibers exhibited a wavy structure. However, treatment with MI-2 induced a more uniform “inearization” or “straightening” of the elastin fibers, which was reversed by cot-reatment with Fer-1 (Fig. 6D). Hematoxylin and eosin (HE) staining revealed that the number of SMCs within the vessel wall following MI-2 treatment was not significantly reduced (Fig. 6E, F). To further characterize the loss of contractility of mouse aortic tissues in response to MI-2 treatment, we measured the expression levels of contractility-related proteins in tissues after wired myography assays. Interestingly, our WB analysis revealed that treatment with MI-2 significantly reduced GPX4 levels, as well as the expression levels of Calponin1 and SM-22, but not SM α-actin (Fig. 6G, H). In summary, our data suggest that MI-2 treatment results in the rapid degradation of contractile proteins, contributing to the loss of contractility.

### MI-2 ameliorates neointimal lesions in C57BL/6J mice

**Fig. 7:**
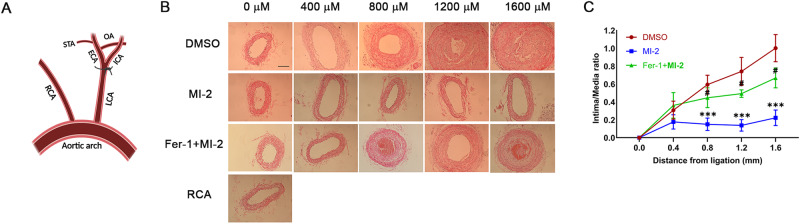
Local application of MI-2 ameliorates neointimal lesions in carotid arteries of C57BL/6 mice. As described in the Methods, a neointima formation model was generated in C57BL/6J mice through complete ligation of the left common carotid artery (LCA). Hydrogel containing MI-2 (10 µM) alone, MI-2 (10 µM) with Fer-1 (50 µM), or the same volume of DMSO, was directly applied to the ligation site. Mice were sacrificed after 3 weeks for analyses of neointima lesions in carotid arteries. **A** Schematic presentation of complete carotid ligation of the LCA, as detailed in the Methods. ECA external carotid artery, ICA internal carotid artery, OA occipital artery, RCA right carotid artery, STA superior thyroid artery. **B** Representative tissue sections (5 µm thickness) from 2 mm proximal to the ligated site were stained with HE for morphometric analyses. Neointima lesions at day 21 after ligation with various treatments were compared at different locations from ligation sites. Scale bar = 100 µm. **C** Cumulative data showing the ratio of intima to lumen area. ***p < 0.01 MI-2 group versus DMSO group; ^#^*p* < 0.05 MI^-^2 +Fer-1 group versus MI-2 group; *n* = 6.

As established above, MI-2 induces ferroptosis in cultured aortic SMCs and ex vivo mouse aortas. We were interested in examining the impact of MI-2 on proliferative diseases in vivo. To do this, we generated neointimal lesions by completely ligating the carotid arteries of C57BL/6J mice, as we have previously described [33, 38] (Fig. 7A). Following ligation, MI-2 was applied to the ligated arteries through perivascular collars, with and without co-treatment using Fer-1. After 3 weeks, mice were sacrificed for neointimal lesion analysis. Our results indicated that MI-2 treatment significantly inhibited neointima formation in the left carotid artery compared to the vehicle control group, and this effect was significantly reversed by co-treatment with Fer-1 (Fig. 7B, C). In conclusion, our results suggest that MI-2 inhibits proliferative vascular disease through the induction of SMC ferroptosis.

### MI-2 inhibits carotid atherosclerosis in ApoE^−/−^ mice in response to partial carotid ligation

**Fig. 8:**
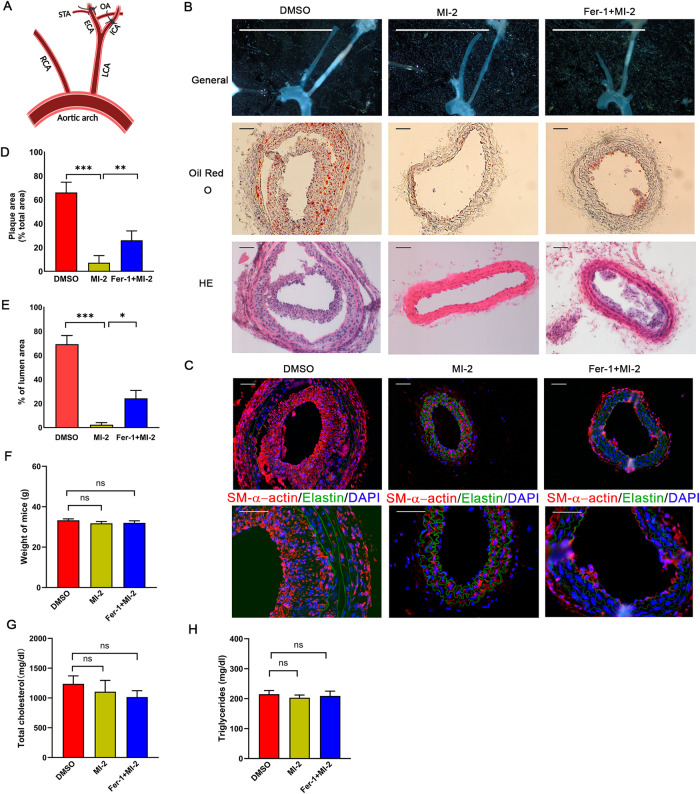
Treatment with MI-2 inhibits carotid atherosclerosis induced by partial carotid artery ligation in ApoE^−/−^ mice. A model of carotid atherosclerosis was generated through partial ligation of the carotid artery of ApoE^−/−^ mice, followed by a high-fat diet and analyses of atherosclerosis in partially ligated carotid arteries, as described in the Methods. Hydrogel containing MI-2 (10 µM) alone, MI-2 (10 µM) with Fer-1 (50 µM), or the same volume of DMSO, was directly applied to the ligation sites. **A** Schematic presentation of partial carotid ligation of the LCA, as detailed in the Methods. **B** After 14 days, mice were sacrificed for analysis of carotid atherosclerosis. Upper panels: Representative photographs of isolated LCAs. Scale bar = 1 cm. Middle panels: Representative tissue sections (10 µm thick) of Oil Red O staining. Lower panels: Representative tissue sections (5 μm thick) of HE staining. Scale bar = 100 μm. **C** Representative tissue sections (10 µm thick) of immunofluorescence staining showing DAPI (blue), elastin (autofluorescence, green), and SM-α-actin (red). Scale bar = 100 µm**. D**, **E** Cumulative data showing the percentage of plaque area (**D**) and the percentage of lumen area (**E**). *p < 0.05; **p < 0.01; ***p < 0.01; *n* = 6-7/group. **F**–**H** At the end of treatment, ApoE^−/−^ mice were sacrificed, and blood samples were collected for analyses of total cholesterol and triglyceride using Cholesterol E and Triglyceride kits, respectively. All mice were analyzed for their body weight (**F**), total blood cholesterol (**G**), and triglyceride levels (**H**). ns not significant; *n* = 6.

It is well-established that neointima formation, characterized by SMC proliferation, plays a critical role in the early stages of atherosclerosis [39]. The observed inhibitory effect of MI-2 on neointima formation prompted us to investigate its impact on the development of atherosclerosis. As reported by Nam et al., partial ligation of the common carotid artery (CCA, Fig. 8A) can rapidly induce carotid atheromas within 2 weeks in ApoE^−/−^ mice on a high-fat diet [40]. After partial ligation, MI-2 was directly applied to the carotid arteries via a perivascular collar, following the protocol described in our neointima formation model. One day after partial carotid ligation, we conducted ultrasound analyses. We observed that the left common carotid artery (LCA) blood flow was significantly decreased compared to that in the right common carotid artery (RCA) (Supplemental Fig. S6A, B). The results from Oil Red O staining showed that the application of MI-2 led to a significant reduction in plaque formation within the LCA, an effect partially reversed by co-treatment with Fer-1 (Fig. 8B–E). In addition, our immunofluorescence results showed that the main component in the plaque is SMCs, which confirmed it mimicked the early stages of atherosclerosis. There was no significant difference in mouse weight, total blood cholesterol, and triglyceride levels among the three groups (Fig. 8F–H).

## Discussion

In this study, we have demonstrated that the pharmacological inhibition of MALT1 by MI-2 induces ferroptosis of vascular SMCs by inhibiting mTOR and activating autophagy. Notably, MI-2 treatment resulted in a rapid loss of contractility in mouse aortas, an effect blocked by co-treatment with Fer-1. Additionally, the application of MI-2 via perivascular collars significantly hindered neointima development in C57BL/6J mice with complete carotid ligation, and atherosclerosis in ApoE^−/−^ mice with partial carotid ligation on a high-fat diet. In conclusion, our results demonstrate that MALT1 inhibition in vascular SMCs leads to ferroptosis resulting in loss of contractility, inhibition of neointima formation, and decrease of atherosclerosis.

To our knowledge, this is the first report showing that MI-2 induces ferroptosis. MI-2 was identified as a small molecule and a novel irreversible inhibitor of MALT1 [6]. Recent evidence has shown that MI-2 can induce apoptosis in several cancer cell lines. For instance, MI-2 significantly decreased anti-apoptotic proteins such as BCL2 and MCL-1 (myeloid leukemia 1) and activated the intrinsic apoptosis pathway in chronic lymphocytic leukemia [41]. Similarly, MI-2 inhibited proliferation and induced apoptosis in T-cell acute lymphoblastic leukemia (T-ALL) cells [20]. However, our results indicate that the cell death induced by MI-2 was not apoptosis but ferroptosis. Multiple pieces of evidence support this conclusion: (1) MI-2-induced cell death was ameliorated by co-treatment with Fer-1 and DFO, two well-known inhibitors of ferroptosis, but not by inhibitors of apoptosis, pyroptosis, or necrosis; (2) MI-2 did not increase cleaved caspase 3 levels; whereas, cleaved caspase 3 levels were increased by 2-methoxyestradiol (2-ME) treatment, as we previously reported [42]; (3) MI-2 treatment time-dependently reduced the expression levels of GPX4 and FTH1, a characteristic change during ferroptosis; (4) MI-2 increased intracellular iron levels, which were reversed by treatment with either Fer-1 or DFO; (5) Erastin, a well-known ferroptosis inducer, mimicked several effects of MI-2 on cultured SMCs, such as increased intracellular iron. Therefore, we conclude that MI-2 treatment induces ferroptosis in cultured vascular SMCs. However, a limitation of our study is that we did not examine the effect of MI-2 on other cell types, even though this effect has been verified in cultured vascular SMCs from rats, mice, and humans.

Importantly, our studies have unveiled the role of autophagy activation in MI-2-induced ferroptosis. We have provided several pieces of data to substantiate this mechanism. First, we noticed the activation of autophagy in vascular SMCs in response to MI-2, as indicated by decreased p62 levels and increased LC3B-II/I ratio. Second, inhibition of autophagy by inhibitors such as 3-MA, Baf A1, CQ, or Atg7 deficiency, ameliorated MI-2-induced ferroptosis. Notably, our observation that Atg7 knockout (KO) in primary cultured mouse aortic SMCs reduces MI-2-induced ferroptosis has phenocopied that observed in other cell types in response to KO of Atg5, Atg7, or Atg13 [29, 32]. It is crucial to note that the impact of autophagy inhibition on MI-2-induced ferroptosis is especially noticeable in the early stages of ferroptosis and under moderate induction conditions. However, this effect significantly diminishes as time progresses or when ferroptosis induction becomes more intense. Nevertheless, this observation aligns with previous studies conducted on erastin-induced ferroptosis [29, 32]. Additionally, our data have shown that treatment with MI-2 inhibited the Akt/mTOR/p70 S6K pathway, further clarifying the mechanism underlying autophagy activation. However, we lack data demonstrating how MI-2 inhibits the Akt/mTOR pathway. In mantle cell lymphoma cells, Jiang et al. reported that MI-2 decreased cell viability, adhesion, and migration by suppressing the PI3K/Akt/mTOR and NF-κB pathways [36]. In addition, increasing evidence suggests a cause-effect relationship between autophagy activation and ferroptosis in other cell types [32, 43, 44]. Also, emerging evidence has demonstrated the interplay between the mTOR and GPX4 signals, modulating autophagy-dependent ferroptosis in several cancer cells [45–48].

It was also reported that the MALT1 paracaspase activity participates in mTOR activation in T cells upon antigen receptor engagement [49, 50]. Moreover, MALT1 inhibition by Z-VRPR-fmk (Z-Val-Arg-Pro-DL-Arg-fluoromethyl ketone) inhibited the phosphorylation of S6 and p70 S6K in T cells [49]. In glioblastoma stem-like cells, mepazine, another MALT1 inhibitor, and MALT1 siRNA were reported to blunt mTOR activation, as assessed through the phosphorylation of Akt, p70 S6K, and S6 ribosomal protein [51]. Hence, existing literature strongly supports the mechanism we unveiled in vascular SMCs. In summary, MI-2-induced autophagy activation is critical in ferroptosis in aortic SMCs. However, further investigation is required to fully understand how MI-2 activates autophagy in vascular SMCs, even though we have shown MI-2 inhibition of the Akt/mTOR/p70 S6K pathway.

MI-2 treatment time-dependently reduced the cleavage of CYLD but did not significantly alter the cleavage of other substrates, such as A20, BCL10, and MCPIP1. This finding has at least two implications. First, MI-2 inhibition of MALT1 may be cell type-specific, i.e., MI-2 inhibition of MALT1 reduces the cleavage of specific substrates depending on the cell type. In endothelial cells, for example, MI-2 treatment increases MCPIP1 [15], an identified MALT1 substrate that was not detected in cultured vascular SMCs in the absence or presence of MI-2. Also, MI-2 treatment did not affect cleavage of other substrates (A20 and BCL10) except CYLD. Note that total MALT1 activities were not measured in all cases represents one of our limitations. Second, reducing CYLD cleavage by MI-2 may mediate autophagy activation and subsequent ferroptosis in vascular SMCs. Several pieces of evidence suggest an interrelationship between CYLD and ferroptosis. First, accumulating evidence has highlighted the vital role of the ubiquitin system enzymes in regulating the sensitivity of ferroptosis, especially in cancer cells [52]. CYLD, a deubiquitinating enzyme, cleaves the lysine 63-linked polyubiquitin chains from target proteins [52]. It is conceivable that decreased cleavage of CYLD by MI-2 inhibition of MALT1 contributes to SMC ferroptosis. In addition, Peng et al. demonstrated that the upregulation of spermatogenesis-associated protein 2 (SPATA2), a member of the tumor necrosis factor (TNF) signaling pathway, and CYLD was accompanied by an increase in ferritinophagy as well as ferroptosis in C57BL/6J mice treated with doxorubicin, which compromised cardiac functions [53]. Whether CYLD cleavage by MALT1 is involved in ferroptosis requires further investigation, such as the overexpression of CYLD and expression of CYLD mutants resistant to MALT1 cleavage.

Our in vivo and ex vivo studies have expanded the significance of the findings observed in cultured SMCs. In our mouse model of neointima formation, perivascular application of MI-2 significantly reduced neointima formation. This effect was partially, but significantly, reversed by co-treatment with Fer-1, confirming a role for ferroptosis induced by MI-2. Moreover, perivascular application of MI-2 significantly reduced atherosclerosis in carotid arteries of ApoE^−/−^ mice fed a high-fat diet, suggesting that induction of ferroptosis in vascular SMCs inhibits neointima formation and the early development of atherosclerosis. Notably, perivascular treatment with MI-2 did not significantly affect mouse body weight and blood cholesterol profiles. The fact that we did not analyze the MI-2 effects on other cell types, such as endothelial cells and inflammatory cells, such as macrophages, represents one of the limitations of our current studies. Nevertheless, our results suggest that MI-2 inhibition of MALT1 may become a promising treatment for proliferative vascular diseases.

In addition, our studies using mouse aortic preparations treated with or without MI-2 not only mirrored the previous finding that treatment with cigarette smoke extract induces ferroptosis of SMCs in mouse aortic tissues with significant morphological changes but also provided functional significance—MI-2-induced ferroptosis causes a rapid loss of SMC contractility. Our WB results demonstrated that MI-2 treatment not only reduced GPX4 levels but also significantly decreased the expression levels of contractile proteins, such as SM-22 and Calponin1, which likely resulted from degradation. Although SM-α-actin levels were not significantly downregulated, the effects of MI-2 on other contractile proteins were likely sufficient to cause the loss of contractility; however, the exact mechanism requires further exploration.

It must be noted that there were two previous studies demonstrating that induction of ferroptosis results in SMC phenotypic conversion from contractile to synthetic SMCs [23, 25]. However, it was unclear how the phenotypic conversion of SMCs was connected with programmed cell death. Changes related to ferroptosis may induce SMC phenotype conversion. Indeed, MI-2-induced ferroptosis rapidly downregulated certain contractile proteins, but we could not observe the occurrence of phenotypic conversion because MI-2 induced ferroptotic cell death. MI-2-induced degradation of contractile proteins in aortic tissue may be a part of the cell death response, but the exact mechanisms still need further clarification. Finally, the loss of smooth muscle contractility and weakening of the arterial wall have been considered the key pathophysiological features in abdominal aortic aneurysm (AAA) and aortic dissection (AD) because of their contribution to subsequent dilation and dissection [54]. Thus, the finding from our ex vivo studies implies a critical role of SMC ferroptosis in the pathogenesis of AAA and AD.

In summary, our studies have revealed a novel ferroptotic effect of MI-2 inhibition of MALT1 on vascular SMCs through the activation of autophagy. These findings suggest that MALT1 could be a potential therapeutic target for proliferative vascular disease.

## Methods and Materials

### Reagents and Antibodies

Collagenase Type II (#9001-12-1), Dulbecco’s Modified Eagle Medium (DMEM)-F12 medium (#2323609), 10000 units/mL penicillin (#15140122), 10000 units/mL streptomycin solution (#15140122), 200 mM L-glutamine (#25030081), Fetal Bovine Serum (FBS; #A4766801), and Hoechst 33342 (#H3570) were purchased from Thermo Fisher Scientific (Ottawa, ON, Canada). Deferoxamine (DFO; #ab120727) and MI-2 (#ab145047) were obtained from Abcam (Cambridge, MA). Ferrostatin-1 (Fer-1; #A13247), Necrostatin-1 (Nec-1; #A11973), and Z-YVAD-fmk (#A16317) were acquired from Adooq Bioscience (Irvine, CA). Erastin (#S7242), Z-VAD-fmk (#S7023), QNZ (EVP4593, #S4902), and 3-Methyladenine (3-MA, #S2767) were procured from Selleck Chemicals (Houston, TX). Bafilomycin A1 (#11038) and chloroquine diphosphate salt (CQ, #C6628) were sourced from Cayman Chemical (Ann Arbor, MI). Sodium chloride (NaCl, #S5886), calcium chloride (CaCl_2_, #C4901), D-(+)-glucose (#D9434), sodium bicarbonate (NaHCO_3_, #S6014), potassium chloride (KCl, #P9333), (R)-(-)-phenylephrine hydrochloride (PE, #PHR1017), rapamycin (#R0395), 4′,6-Diamidine-2′-phenylindole dihydrochloride (DAPI, # 10236276001) and propidium iodide (PI; #P4170) were purchased from Sigma-Aldrich Canada (Oakville, ON, Canada). Primary antibodies for caspase 3 (#14220), cleaved-caspase 3 (#9664), p62 (#5114), LC3B (#83506), Atg7 (#8558), MALT1 (#2494), A20 (#5630), p-mTOR (#5536), mTOR (#2983), p-Akt (#4046), Akt (#4691), p-p70 S6K (#97596), p70 S6K (#9209), GAPDH (#5174), goat anti-rabbit secondary antibody (#7074), and goat anti-mouse secondary antibody (#7076) were purchased from Cell Signaling Technology (Whitby, ON, Canada). Calponin1 (#EP798Y), SM22 (#MABT167), β-actin (#A5441), and SM-α-actin (#A2547) were purchased from Sigma-Aldrich Canada (Oakville, ON, Canada). GPX4 (#ab125066), FTH1 (#ab75973), and TOM20 (#ab186735) antibody were purchased from Abcam. BCL10 (#sc-5273) and cylindromatosis 1 (CYLD, #sc-74435) antibodies were purchased from Santa Cruz Biotechnology (Santa Cruz, CA). Total cholesterol kits (#999-02601) and triglyceride kits (#290-63701) were acquired from FUJIFILM (Lexington, MA).

### Cell culture

Human smooth muscle cells (SMCs, ATCC CRL-199) were cultured and maintained in DMEM-F12 medium supplemented with 100 U/mL penicillin, 100 U/mL streptomycin, and 10% FBS. Primary rat SMCs were isolated from the media of Sprague-Dawley (SD) rat aortas as previously described [55]. Briefly, the aortas were carefully dissected, with the surrounding fat tissues and the adventitia removed and the intima scraped off. The aortas were then sectioned into approximately 1 mm^3^ pieces, followed by explantation for approximately 2 h before adding DMEM-F12 medium supplemented with 20% FBS. After reaching sub-confluence, the cells were subcultured using DMEM-F12 medium enriched with 10% FBS and penicillin/streptomycin.

Primary mouse SMCs were similarly cultured from aortas of C57BL/6J mice (Charles River Laboratories, Quebec, QC, Canada) or Atg7 floxed mice purchased from RIKEN BioResource Center (Ibaraki, Japan), as previously described [33]. In brief, the aortas were cut into small, approximately square sections measuring 1–2 mm with the adventitia removed. They were then digested with 1.2 mg/ml type II collagenase and incubated in a standard tissue culture environment at 37 °C, 5% CO_2_, for 4–6 h. A complete culture medium was subsequently added to halt digestion, and the samples were centrifuged at 1000 × *g* for 5 min. The cells were resuspended and cultured with DMEM/F12 containing 10% FBS.

Immunofluorescence staining was performed on isolated SMCs, and the content of α-SMA (SM α-actin) positive cells exceeding 80% suggested the successful extraction of SMCs. Cells showed no mycoplasma contamination. Experiments were performed using primary SMCs from passages 4 to 10. Treatments were administered when cells reached approximately 70% confluency.

### PI and Hoechst staining

Cells were stained using a final concentration of 5 µg/mL of both propidium iodide (PI) and Hoechst 33342. The staining solution, which was a mixture of these dyes dissolved in phosphate-buffered saline (PBS), was added directly to the culture medium post-treatment. Following this, cells were incubated for 10 min at 37 °C in a humidified atmosphere of 95% air and 5% CO_2_. Subsequently, the staining medium was discarded, and the cells were washed three times with PBS. Analysis was carried out using fluorescence microscopy.

### MTT assay

Cell viability was assessed using 3-(4,5-dimethylthiazol-2-yl)-2,5-diphenyltetrazolium bromide (MTT) with slight modifications [56]. A cell proliferation kit (MTT, # 11465007001) was purchased from Sigma-Aldrich Canada (Oakville, ON, Canada). This assay measures the transformation of MTT into purple formazan crystals by the enzyme succinate dehydrogenase found within the intact mitochondria of active cells. Rat aortic SMCs were plated into 96-well plates at a density of 1 × 10^4^ cells per well in triplicate and allowed to incubate for 12 h. Following treatment, 10 μL of a 5 mg/mL MTT solution was added to each well, and the cultures were further incubated for 4 h. After that, 100 μL of solubilization solution (10% SDS in 0.01 M HCl) was added to each well. After overnight incubation at 37 °C and 5% CO_2_ in a humidified environment, the absorbance was recorded at 595 nm using an iMark™ Microplate Reader (Bio-Rad Canada, Mississauga, ON, Canada).

### Iron assay

Intracellular ferrous iron levels were evaluated using an Iron Colorimetric Assay Kit obtained from ScienCell (#8448, Carlsbad, CA). Following the manufacturer’s instructions, cells were centrifuged at 13,000 × *g* for 10 min at 4 °C to obtain the supernatant for the assay. A 50-μL sample was then incubated with 50 μL of assay buffer in a 96-well microplate for 30 min at 25 °C. Subsequently, the sample was incubated with 200 μL of reagent mix in the dark for an additional 30 min at 25 °C. Absorbance was measured at 590 nm using an iMark Microplate Reader.

### Western blot (WB) analysis

Cultured rat SMCs, subjected to various treatments, were washed three times with cold PBS, then lysed with RIPA buffer (containing 150 mM sodium chloride, 1.0% NP-40, 0.5% sodium deoxycholate, 0.1% sodium dodecyl sulfate (SDS), and 50 mM Tris with a pH of 8.0, as well as protease inhibitors) for 30 min on ice. This was followed by protein extraction and measurement of protein concentration using a BCA Protein Assay Kit (Bio-Rad Canada, Mississauga, ON, Canada). The total protein concentration was determined using an iMark Microplate Reader. An equal amount of protein from each sample was separated by 7.5%, 10% or 12% SDS-polyacrylamide gel electrophoresis (SDS-PAGE) and then transferred to nitrocellulose filter membranes. The membranes were blocked with 10% non-fat dry milk, then incubated overnight at 4 °C with primary antibodies. Subsequently, the membranes were incubated with secondary antibodies for 1 h at room temperature, followed by three washes with tris-buffered saline with 0.1% Tween 20 detergent. The abundance of immunolabeled proteins was detected using enhanced chemiluminescence (ECL) and quantified using an ImageQuant LAS-4000 (GE Healthcare, Mississauga, ON, Canada) and Gel-Pro Analyzer version 4.0 software (Media Cybernetics, Maryland, MD).

### Immunofluorescence staining

Cultured rat SMCs were seeded onto poly lysine slides and subjected to various treatments, followed by fixation with 4% paraformaldehyde (PFA) diluted in PBS for 15 min. The cells were then permeabilized with 0.25% Triton X-100 and blocked with 5% PBS-BSA (bovine serum albumin) before being incubated with primary antibodies for 1 h. After washing with PBS, cells were incubated with Alexa Fluor-conjugated secondary antibodies for 45–60 min. Images were captured with a fluorescence microscope and quantified by Image-Pro Plus software (Version X, Media Cybernetics, Silver Springs, MD)

### Organ culture and myography

Male C57BL/6J mice, aged 6 to 8 weeks and weighing between 19 and 20 g, were sourced from Charles River Laboratories (Quebec, QC, Canada). All animal studies were approved by the Institutional Animal Care and Use Committees at the University of Calgary and were conducted in accordance with the guidelines set by the US National Institutes of Health. The aortic rings, approximately 2 mm long, were dissected and mounted in a 5 mL myograph chamber for isometric tension recording. The aortic ring was perfused with a 1 mg/mL solution of sodium deoxycholate in saline for 30 s to remove the vascular endothelium, as described previously [57, 58]. The aortic rings in Krebs solution (114 mM NaCl, 4.7 mM KCl, 0.8 mM KH_2_PO_4_, 1.2 mM MgCl_2_, 11 mM d-glucose, 25 mM NaHCO_3_, and 2.5 mM CaCl_2_, with a pH of 7.4), which was bubbled with 95% air and 5% CO_2_, were maintained at a resting tension of 0.5 g. Aortic rings were pre-treated with dimethyl sulfoxide (DMSO) or Fer-1 (10 µM) for 90 min, followed by the addition of MI-2 (2.5 µM) for another 90 min and subsequent measurement of their contractile responses to 50 mM KCl and 1 μM PE, respectively. The tissues were washed a minimum of three times between applications of the contractile agonist and the commencement of the next response, which was initiated 45–60 min later. Upon completion of the experiment, the tissues were fixed with 4% PFA and embedded in paraffin. Serial sections, 5 μm thick, were then prepared for examination via Elastic Van Gieson staining and Hematoxylin and Eosin (HE) staining.

### Neointima formation in C57BL/6J mouse carotid arteries

C57BL/6J male mice, 8 weeks of age, were housed in a regulated environment, with light and temperature cycles maintained at 12 h of light and 12 h of darkness at a temperature of 22 ± 2 °C. The mice had *ad libitum* access to food and water. Each mouse underwent a complete carotid artery ligation, in which the left carotid artery was dissected and ligated proximal to its bifurcation, leaving the right carotid artery as a control. The surgically treated mice were randomly divided into three groups: MI-2, MI-2 plus Fer-1, and control, which received an equal volume of DMSO. The investigator was not blinded to the group allocation.

MI-2 and Fer-1 were dissolved in 25% F-127 Pluronic gel (Sigma-Aldrich) and applied to the ligated segment of the carotid artery. Their concentrations were 10 times higher than those used for in vitro studies (10 μM for MI-2 and 50 μM for Fer-1). Fourteen days post-ligation, the left carotid arteries were harvested, fixed with 4% PFA for 12 h at 4 °C, and subsequently embedded in paraffin. Serial paraffin sections of 5 μm thickness, encompassing the region proximal to the ligation site, were subjected to HE staining.

### Partial carotid ligation and atherosclerosis model in ApoE^−/−^ mice fed a high-fat diet

Four-month-old ApoE^−/−^ male mice were randomly allocated to vehicle control (DMSO), MI-2, or MI-2 plus Fer-1 treatment groups (the investigator was not blinded to the group assignments). A partial carotid ligation was performed as previously described [38, 59]. The anesthesia protocols utilized were identical to those outlined for complete ligation. Briefly, the three branches of the LCA—the left external carotid artery (ECA), left internal carotid artery (ICA), and occipital artery (OA)—were ligated using a 9–0 Ethalon suture, leaving the superior thyroid artery (STA) intact. Post-ligation, all ApoE^−/−^ mice received either a perivascular application of MI-2 (10 µM) or MI-2 (10 μM) plus Fer-1 (50 μM) using 25% hydrogel at the ligation site or a control treatment of 25% hydrogel with DMSO. The LCA patency was confirmed in all mice using ultrasound the day after ligation and reconfirmed 2 weeks post-ligation. All mice were fed a high-fat atherogenic diet (21% milk fat, 1.25% cholesterol). After 2 weeks, mice were euthanized in a CO_2_ chamber, blood samples were collected, and arteries were perfused to remove residual blood. Arterial tissues were then fixed with PFA, embedded in Optimal Cutting Temperature compound (OCT, -20 °C), and sectioned (10-μm-thick) for Oil Red O (Abcam #ab150678, Cambridge, MA).

### Statistical analysis

The data represent at least three independent experiments unless otherwise stated. Data are presented as mean ± standard error of the mean (SEM). Statistical analysis was conducted using IBM SPSS Statistics version 25.0 (IBM, Chicago, IL). This analysis included either a one-way analysis of variance (ANOVA) or an unpaired two-tailed Student’s *t* test. A P-value of less than 0.05 was considered statistically significant.

### Supplementary information

Supplementary Fgures

Supplementary Figure Legend

## Data Availability

Preliminary data may be obtained by requesting it from the corresponding author.
