# Supplementary material for: Pharmacological inhibition of MALT1 (mucosa-associated lymphoid tissue lymphoma translocation protein 1) induces ferroptosis in vascular smooth muscle cells
Source: Cell Death Discov. 2023 Dec 15;9:456. doi: 10.1038/s41420-023-01748-9 (PMC10721807; doi:10.1038/s41420-023-01748-9)
Supplement: Supplementary file 1 — Supplementary Fgures [file 41420_2023_1748_MOESM1_ESM.pdf]

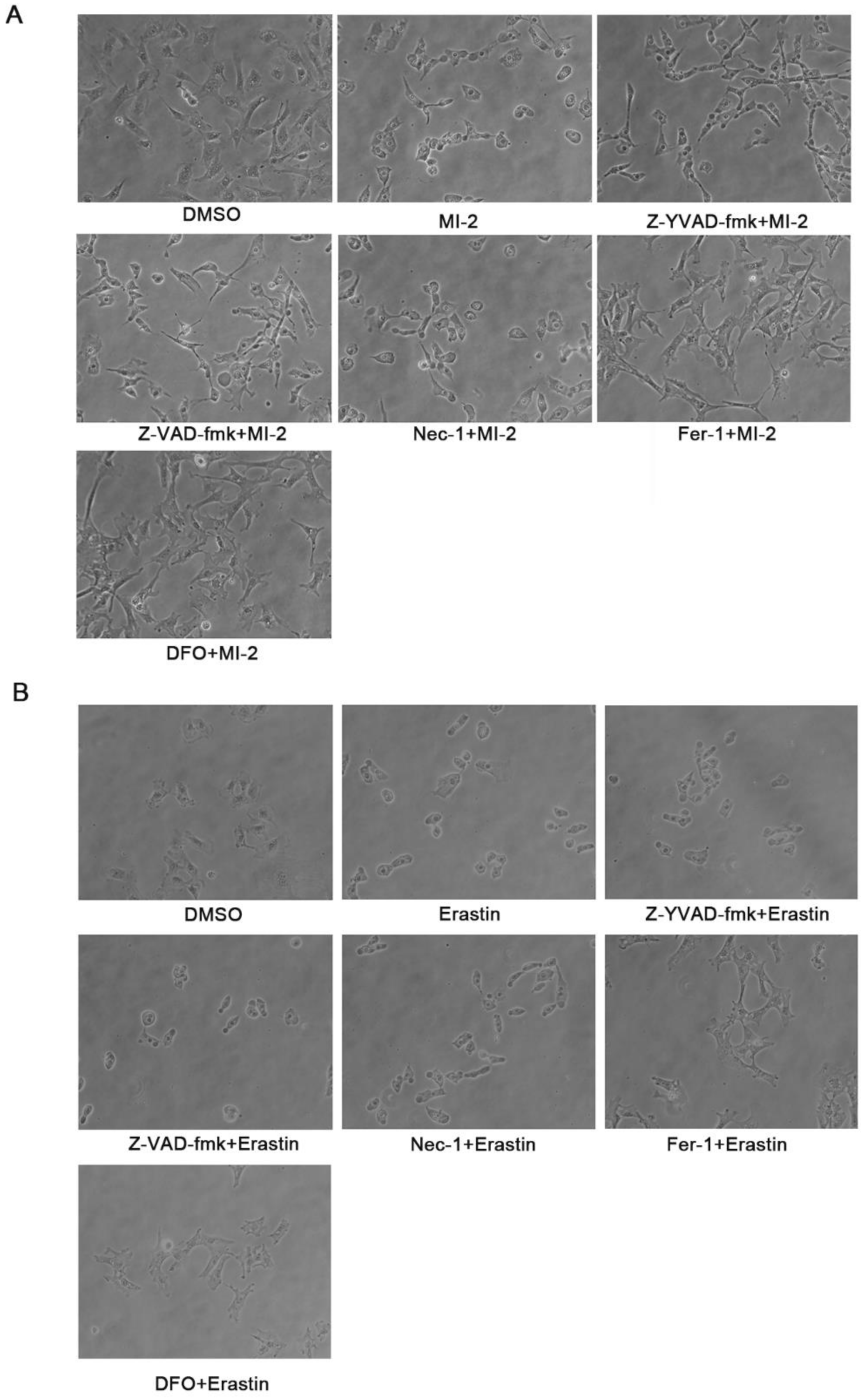

Supplementary Fig. 1

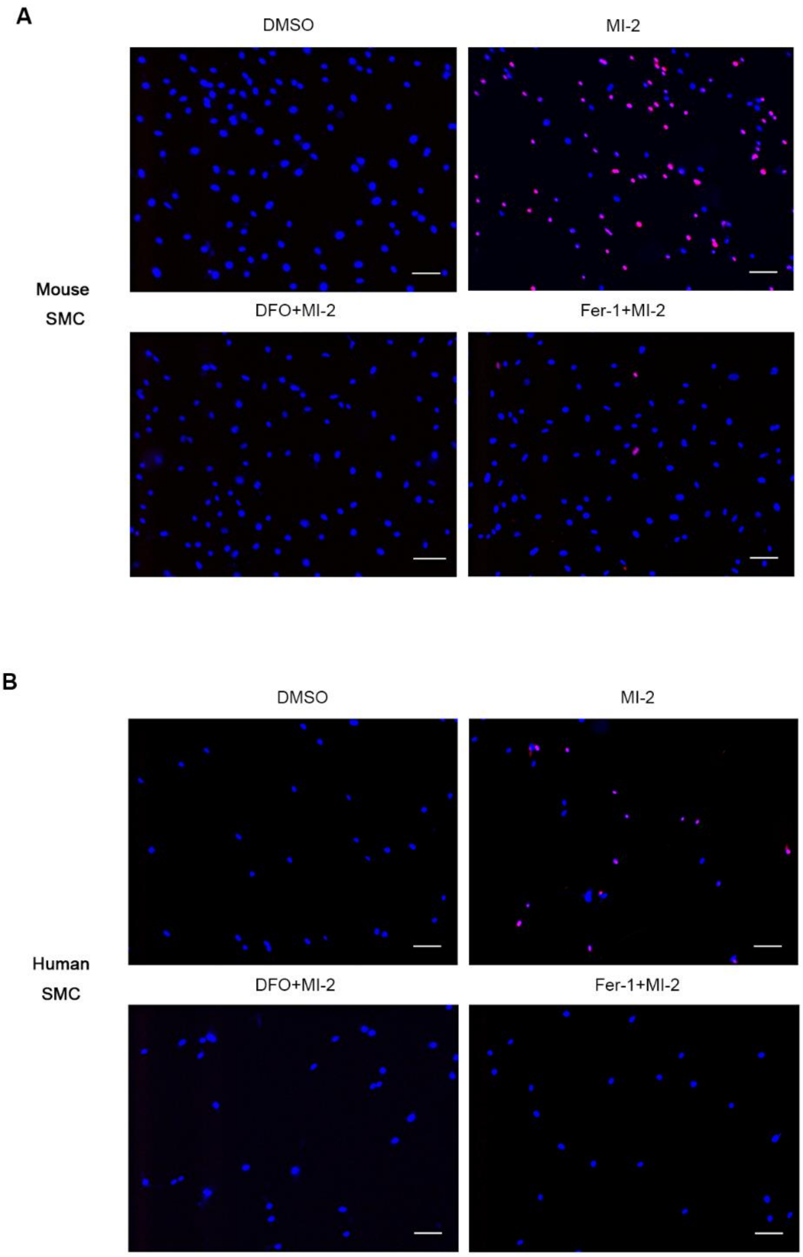

Supplementary Fig. 2

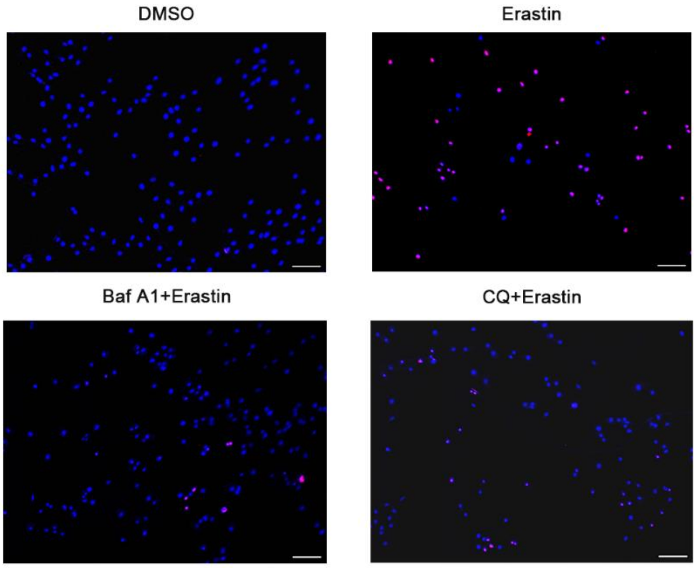

Supplementary Fig. 3

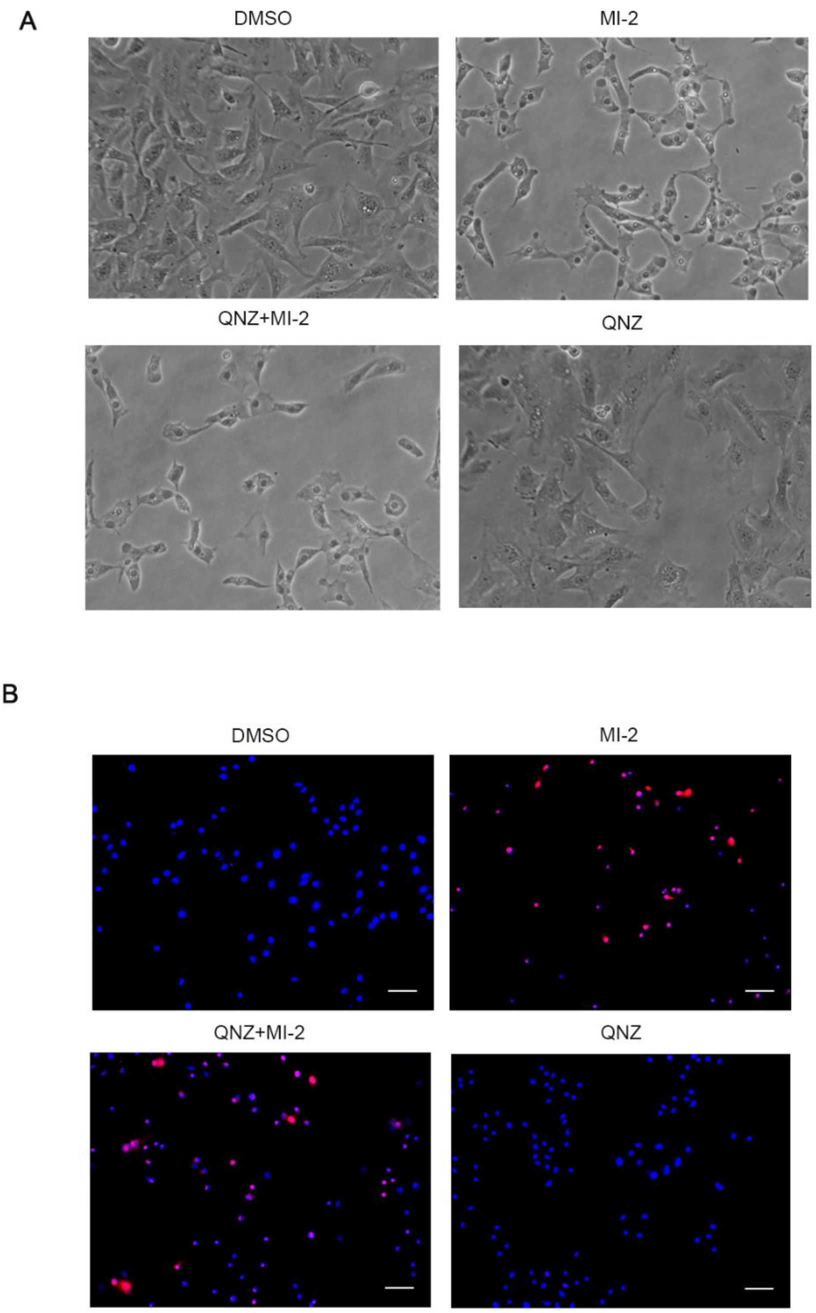

Supplementary Fig. 4

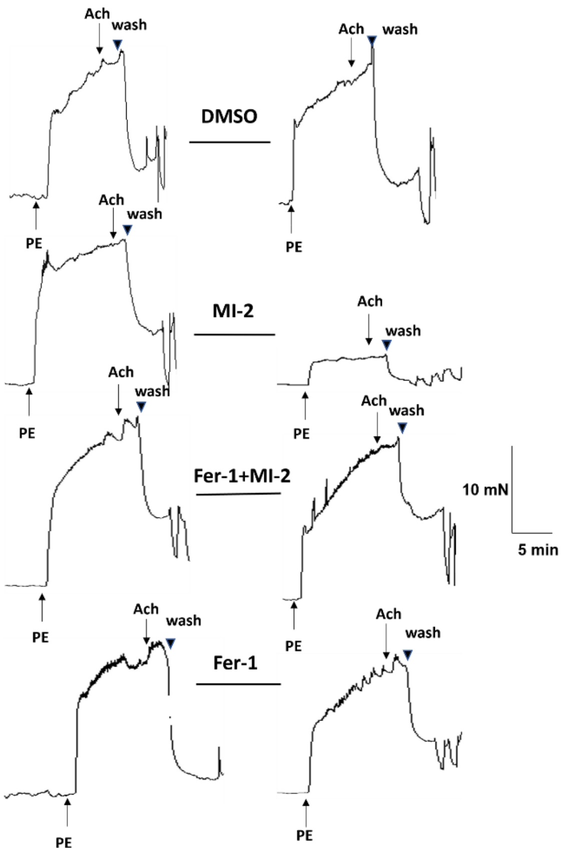

Supplementary Fig. 5

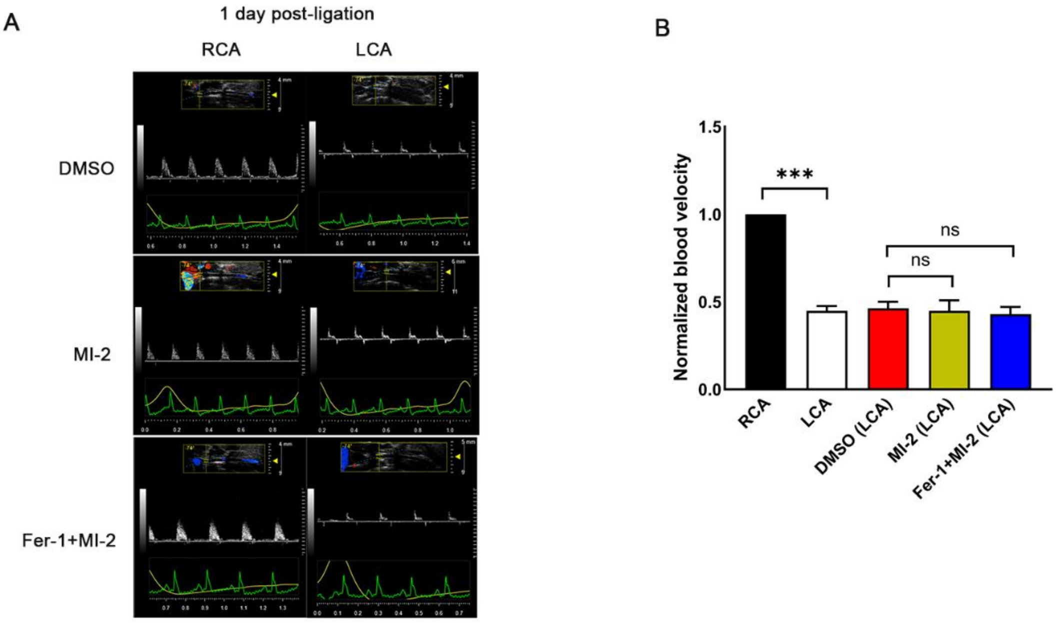

Supplementary Fig. 6
