# Supplementary material for: Pharmacological inhibition of MALT1 (mucosa-associated lymphoid tissue lymphoma translocation protein 1) induces ferroptosis in vascular smooth muscle cells
Source: Cell Death Discov. 2023 Dec 15;9:456. doi: 10.1038/s41420-023-01748-9 (PMC10721807; doi:10.1038/s41420-023-01748-9)
Supplement: Supplementary file 2 — Supplementary Figure Legend [file 41420_2023_1748_MOESM2_ESM.docx]

**Supplementary Fig. 1 Effect of MI-2 or erastin on cellular morphology with or without different inhibitors.** **A and B:** Representative light microscopic images showing treatment with MI-2 (**A**, 1 µM) or erastin (**B**, 5 µM) in primary rat SMCs for 24 hours in the presence or absence of different inhibitors. Magnification 100 $\times$.

**Supplementary Fig. 2. MI-2 induces ferroptosis in mouse or human aortic SMCs**. Representative images showing primary cultured mouse (**A**) and human (**B**) aortic SMCs were treated with MI-2 (1 µM) in the absence or in the presence of Fer-1 (5 μM) for 6 hours, followed by PI-Hoechst co-staining. Scale bar = 100 μm.

**Supplementary Fig. 3** **Erastin-induced cell death is inhibited by autophagy inhibitors. Representative micrographs.** Rat aortic SMCs were treated with 5 µM erastin in the presence or absence of Baf A1 (100 nM) or CQ (50 µM) for 6 hours, followed by PI-Hoechst co-staining. Scale bar = 100 μm.

**Supplementary Fig. 4 NF-κB inhibitor cannot reverse MI-2-induced ferroptosis. A:** Representative light microscopic images showing treatment of MI-2 (1 µM) in primary rat SMCs for 6 hours in the presence or absence of QNZ (NF-κB inhibitor, 1 µM). Magnification 100 $\times$. **B:** Representative immunostaining images showing treatment of MI-2 (1 µM) in primary rat SMCs for 6 hours in the presence or absence of QNZ (1 µM), followed by PI-Hoechst co-staining. Scale bar = 100 μm.

**Supplementary Fig. 5** Representative tracings showing PE responses of tissues before and after treatment with MI-2 (2.5 µM) in the presence of Fer-1 (10 µM) pre-treatment.

**Supplementary Fig. 6 A:** Representative ultrasound images showing flow velocity profiles to confirm that partial ligation reduced blood flow without complete blockage on the day after LCA partial ligation in all mouse groups. **B:** Cumulative data showing the blood velocity on day 1 after surgery. ***p<0.001; ns: not significant; n=6.
